# Airborne Bacteria Are Influenced by Surrounding Soil, Litter and Vegetation at a Forest Campsite

**DOI:** 10.1111/1758-2229.70427

**Published:** 2026-10-07

**Authors:** Keiichi Kimura, Natsuko I. Kondo, Nobuyoshi Nakajima, Hiroshi C. Ito, Taku Kadoya, Kazuaki Tsuchiya

**Affiliations:** ^1^ Social Systems Division National Institute for Environmental Studies Tsukuba Ibaraki Japan; ^2^ Biodiversity Division National Institute for Environmental Studies Tsukuba Ibaraki Japan

**Keywords:** aerobiome, biodiversity hypothesis, environmental DNA, leaf litter, soil bacteria

## Abstract

Contact with natural environments is increasingly linked to human well‐being, yet the environmental sources shaping forest airborne microbiota remain poorly resolved. Here, we examined the relationship between the forest aerobiome and nearby ground reservoirs, focusing on litter and surface soil in a recreational forest. Bacterial communities were compared among air, litter and soil at a campsite spanning two vegetation types: a secondary broadleaf forest with diverse understory and a conifer plantation with sparse understory. Air was continuously sampled for 1 week using membrane filtration, and litter and surface soil were collected adjacent to each air sampler. We identified 6146 amplicon sequence variants across 19 phyla. Habitat (air, litter, soil) was the primary driver of community composition. Airborne communities were significantly distinct from both litter and soil and showed lower diversity. Although dominant higher taxa were shared across habitats, airborne assemblages were compositionally distinct at the genus and amplicon sequence variant levels, whereas litter and soil were more similar to each other. Nonetheless, substantial genus‐level overlap between air and ground habitats was detected in both vegetation types. These findings indicate that litter and soil act as important, but incomplete, environmental reservoirs of forest airborne bacteria in recreational settings.

## Graphical abstract

Airborne bacterial communities in a recreational forest were compositionally distinct from litter and surface soil at the ASV level, as shown by NMDS ordination. Yet partial overlap with ground habitats indicates that local litter and soil act as important, but incomplete, reservoirs shaping the forest aerobiome across contrasting vegetation types.

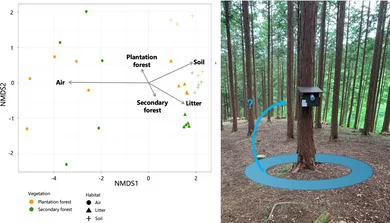

## 1 Introduction

Human well‐being is closely associated with contact with nature, and microbial exposure is increasingly recognized as a key mechanism by which natural environments may promote health. The ‘biodiversity hypothesis’ posits that reduced contact with diverse environmental microbiota can elevate the risk of allergic diseases (Haahtela 2019; Hanski et al. 2012). Consistent with this hypothesis, exposure to a broader range of environmental microbiota can modify the human microbiome (Selway et al. 2020) in ways that may subsequently influence immune development, particularly during childhood (Heederik and Von Mutius 2012). Previous research focused on direct contact with soil or other surfaces as sources of environmental bacteria (Hanski et al. 2012; Roslund et al. 2020). However, during nature experiences such as walking, hiking and camping, people are continuously exposed to the near‐surface atmosphere. Airborne bacteria can be detected despite the poor nutrient availability and intense ultraviolet radiation (Smets et al. 2016), although bacterial viability may decline rapidly after aerosolization (Šantl‐Temkiv et al. 2017). Recent studies suggest an association of exposure to outdoor airborne bacterial communities with variation in the human microbiome (Brame et al. 2024; Lashus et al. 2026; Selway et al. 2020). These findings have generated increasing interest in the aerobiome, or the microbiome of the air, as a critical interface between ecosystems and human health (Robinson and Breed 2023).

A growing body of research indicates that terrestrial habitats are primary sources of airborne bacterial assemblages, although the specific pathways and health implications remain unclear. Microbial communities differ among habitats such as soil and atmosphere, because each environment imposes distinct constraints on resources and physicochemical conditions (Baldrian 2016). In forests, soil and the litter layer are major reservoirs of bacterial biomass (Baldrian 2016), supported in part by carbon released through fungal bioactivity (Smets et al. 2016) and may contribute microbes to the atmosphere. Robinson et al. (2020) demonstrated vertical stratification of the near‐surface urban aerobiome and identified soil as a key contributor to its taxonomic diversity. Robinson et al. (2021) extended this analysis to additional land‐cover types. Urban aerobiome studies have also shown that vegetation types influence the composition of the airborne community (Mhuireach et al. 2016, 2021). Despite these advances, studies directly linking airborne and soil bacterial communities remain limited. The litter layer, a critical interface between soil and atmosphere, has also received little attention as a potential source of airborne bacteria through processes such as wind suspension (Walters et al. 2022). Although litter can often harbour bacterial communities that differ from those in soil (Baldrian 2016), studies examining the similarity between ground habitats and airborne bacterial composition (Robinson et al. 2020, 2021) have not distinguished litter from soil. In addition, most research has focused on urban green spaces (Mhuireach et al. 2016, 2021; Robinson et al. 2020, 2021), whereas peri‐urban or rural recreation sites with semi‐natural environments, which are important destinations for urban residents, remain underrepresented (Aota et al. 2025; Lashus et al. 2026).

This study investigated bacterial communities in air, litter and soil within a forested recreation area in Japan. Adopting aerobiome study designs that compare proximate sites and contrasting vegetation structures (Mhuireach et al. 2021; Robinson et al. 2021), we sampled airborne bacteria alongside litter and surface soil samples as potential terrestrial sources. We sampled two forest types within the same landscape to determine whether relationships between communities are consistent across different vegetation contexts. The objectives were to compare bacterial diversity and community composition among air, litter and soil; to assess whether these compartments share taxa in patterns indicative of terrestrial source contributions to airborne communities; and to evaluate how these patterns differ between forest types used for outdoor recreation. We hypothesized that community structure would vary across habitats and forest vegetation types, and that if dispersal from litter and soil is an important process shaping the aerobiome, airborne communities would partially overlap with taxa found in litter‐ and soil‐associated communities, with consistent linkages detectable across forest vegetation types.

## 2 Materials and Methods

### 2.1 Study Site

The study site consisted of two forest plots in Motegi, eastern Japan (36°53′23″ N, 140°22′54″ E), located within Hello Woods, a camping and outdoor recreation area. This site has been managed to support recreational use and biodiversity conservation for more than two decades, and was designated as an Other Effective Area‐based Conservation Measure site by the Ministry of the Environment in 2023 (Ministry of the Environment of Japan 2025). The plots were placed within a range of ~200 m, comparable to or smaller than the spatial extent examined in previous studies (Robinson et al. 2020, 2021). This approach aimed to reduce variation in airborne bacterial assemblages attributable to broader landscape‐scale atmospheric differences. Both plots have similar topographic and soil conditions but differ in dominant tree composition and human use. They are situated on hills at ~200 m above sea level, characterized by Andosols, and face southwest. The surrounding landscape is predominantly forested hilly terrain, with rice paddies, croplands and small settlements distributed along valley bottoms. One plot is secondary forest derived from traditional rural coppice woodlands, with a canopy dominated by deciduous broadleaf trees, primarily *Quercus serrata* , *Quercus acutissima* and *Cerasus jamasakura*. It supports a well‐developed understory, including *Pleioblastus chino* and diverse forest‐floor vegetation, and walking trails. The other is an evergreen conifer plantation dominated by *Chamaecyparis obtusa* that was established and previously managed for timber production. It has little to no understory vegetation and is now used mainly for camping. To provide a reference for airborne bacterial communities outside the forest environment, we also collected air samples at an adjacent parking area.

### 2.2 Sampling Design

We made samplers to collect airborne bacteria (Figure 1A,B). A mini pump (MP‐Σ100HNII, Sibata) drew air through a filter funnel equipped with a 0.2‐μm‐pore‐size cellulose nitrate membrane filter (*ø* = 47 mm, Thermo Fisher Scientific), which trapped bacteria. The devices were sheltered in a wooden box mounted on five trees in each of the three sampling sites at 1.5 m from the ground. The battery‐powered devices ran continuously from 1 to 7 August 2024. The membrane filters were collected on 7 August 2024 and kept on ice during transportation to the laboratory and then stored at −20°C until analysis. Air was sampled at 1.5 L/min; the average total volume of the sampled air was 12,652 L per device. It was necessary to discard the sample collected from one of the devices in the parking area because of overheating of the pump. Thus, the total number of bacterial samples was 14.

**FIGURE 1:**
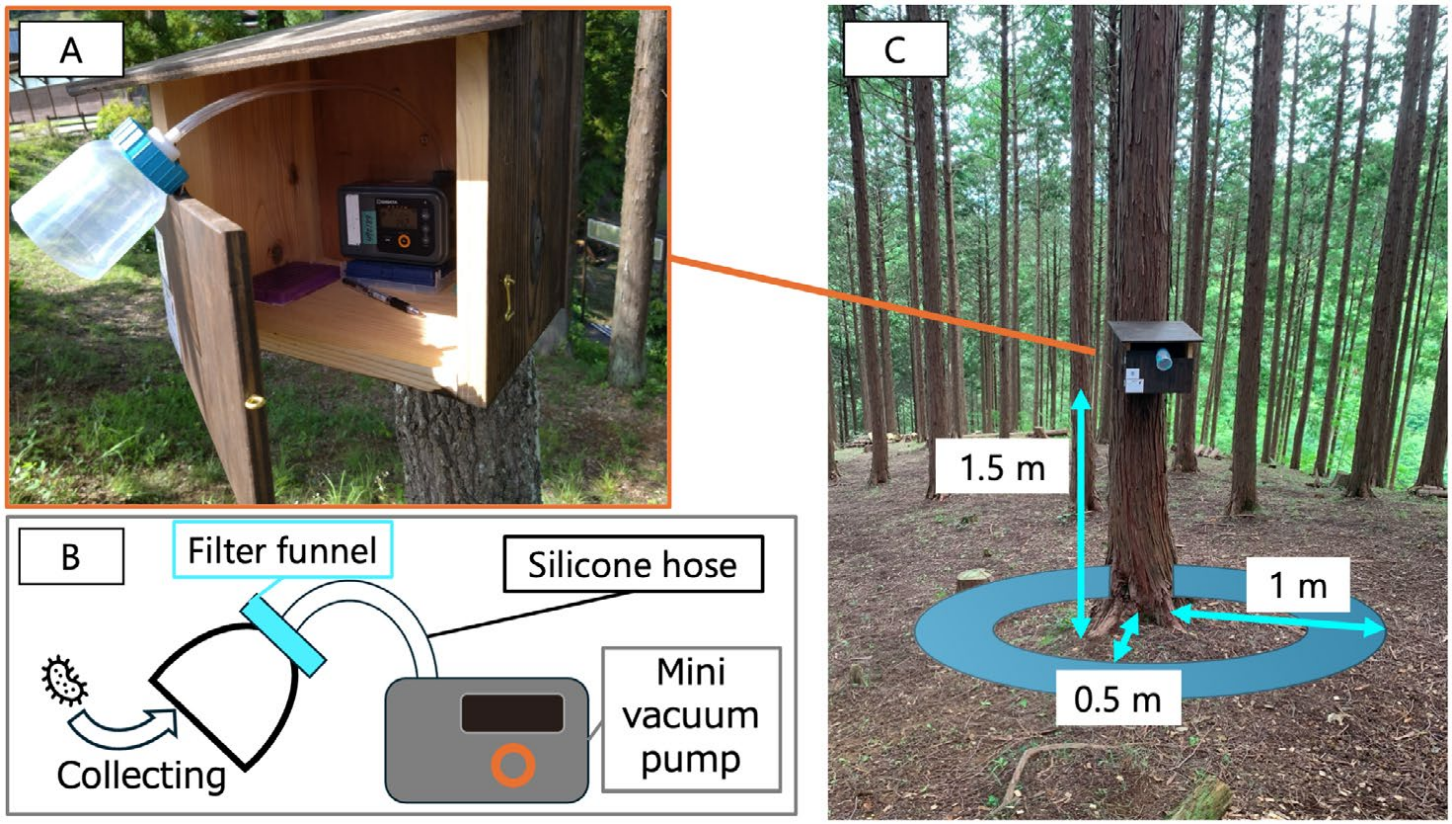
Design for sampling airborne bacteria, soil and litter. (A) Close‐up of the air‐sampling device mounted on a tree at 1.5 m above ground level. (B) Schematic of the sampler, where a battery‐powered vacuum pump draws air through a filter funnel containing a cellulose nitrate membrane filter. (C) Overview of the sampler tree and adjacent ground‐sampling area. Three soil and three litter subsamples are collected within the blue 0.5‐m‐wide ring, positioned 0.5 m from the trunk, and pooled by sample type.

We also collected soil and litter samples within a 0.5‐m‐wide ring spaced 0.5 m away from the trunk of each sampler tree (Figure 1C). The inner 0.5 m was omitted because that was where we had stood to mount the sampler. We placed a litter sample in a 50‐mL centrifuge tube and the upper 5 cm of soil in another tube, collecting three of each per tree, which were then bulked. These samples were held at ambient temperature during transportation and at −80°C in the laboratory until analysis. In total, we collected 10 soil and 10 litter samples from the two forest plots. We did not collect samples from the parking area, which was paved and had flower beds with almost no litter.

### 2.3 DNA Extraction and 16S rRNA Metabarcoding

Airborne DNA was extracted from the membrane filter with a DNeasy Blood and Tissue Kit (Qiagen) and eluted in 100 μL of buffer AE according to the manufacturer's instructions. Soil and litter DNA was extracted with a DNeasy PowerSoil Pro Kit (Qiagen). Each sample was placed in a mortar and ground in liquid nitrogen, then 200 μg of ground sample was transferred to a microtube and further homogenized in TissueLyser with a 5‐mm zirconium bead and buffer (Qiagen). Subsequent procedures followed the manufacturer's protocol, and DNA was finally eluted in 100 μL of buffer AE.

For bacterial metabarcoding, we performed amplicon sequencing targeting the V4 region of the 16S rRNA gene. The V4 region was amplified using Q5 High‐Fidelity DNA Polymerase (New England Biolabs; NEB) with the primer set 515F/806R (Caporaso et al. 2011). The PCR product (~300 bp) was purified with AMPure XP (Beckman Coulter). Illumina adapters and indices were then added with an NEBNext Ultra II DNA Library Prep Kit for Illumina (NEB) together with the NEBNext multiplex Oligos for Illumina (Dual Index Primers Set 1; NEB) following the manufacturer's protocol. The PCR products of each sample were combined in equal volumes and purified twice with AMPure XP. The resulting library was quantified on a TapeStation 4150 electrophoresis platform (Agilent) and then sequenced on a MiSeq sequencer (Illumina) using a MiSeq Reagent Kit v. 3 (600 cycles).

From the raw R1 and R2 sequences (i.e., forward and reverse paired‐end reads), primer sequences were removed in Cutadapt v. 2.8 (Martin 2011) with default settings. Amplicon sequence variants (ASVs) were inferred independently from the R1 and R2 reads in the R package DADA2 v. 1.24.0 (Callahan et al. 2016), with the functions *dada* and *filterAndTrim* for preliminary quality filtering under default settings, and the resulting ASVs were concatenated. ASVs of 240 to 320 bp in length were retained for analysis. Chimeric ASVs were removed with the *removeBimeraDenovo* function of DADA2, and potential contaminants were removed with the R package decontam v. 1.16.0 (Davis et al. 2018). To reduce false positives caused by potential PCR errors and contaminants (see also Nearing et al. 2018), we excluded ASVs with < 8 reads across all samples, in accord with the default threshold value 8 for the ‘‐minsize’ option in another widely used ASV detection tool, UNOISE3 (Isabwe et al. 2019). The taxonomic classification was assigned to resulting ASVs from the SILVA (v. 138.2) database by DADA2 (Callahan 2024). Following taxonomic assignment, only bacterial ASVs were retained for subsequent analyses. A total of 1,541,939 sequences were obtained from all 34 samples. We then rarefied each sample to 9544 sequences (minimum) for downstream analyses (Robinson et al. 2020; Figure S1). The sequence data are deposited in the DDBJ Sequence Read Archive under accession number DRR1053300‐DRR1053333 (Bioproject: PRJDB42365).

### 2.4 Statistical Analyses

To characterize similarities and differences in bacterial taxa among habitats and vegetation types, we summarized sequence and ASV counts at each taxonomic level. At higher taxonomic levels (phylum and class), only taxa with a relative abundance of > 1% within each sample were included, following previous studies (Archer et al. 2023; Robinson et al. 2020). At the genus level, relative abundances were calculated for all taxa, including low‐abundance genera. To understand the contribution of low‐abundance genera, we divided genera into major and minor groups on the basis of previous studies (Robinson et al. 2020; Selway et al. 2020), with minor genera defined as having a relative abundance of < 1% (Robinson et al. 2020). Furthermore, Venn diagrams were generated at the phylum, class, order, family, genus and ASV levels to identify taxa shared among habitats and vegetation types.

We used the Bray–Curtis dissimilarity index for non‐metric multidimensional scaling (NMDS). We used NMDS analyses to visualize the relative effects of the vegetation and habitat types on microbial communities. Vectors and centroids of the variables were fitted into NMDS plots. The significance of observed differences among bacterial communities among habitats or vegetation types was determined by PERMANOVA with Bray–Curtis similarity after 9999 permutations. PERMANOVA was used to compare bacterial community composition among the six combinations of vegetation type (plantation and secondary forests) and habitat type (air, litter and soil), under the null hypothesis that community composition did not differ among these groups. We calculated two Shannon–Weaver diversity indices based on ASV number: one for all bacteria and the other restricted to Gammaproteobacteria class. We specifically focused on and analyzed Gammaproteobacteria because the diversity of this class in the environment has often been cited in previous studies as being associated with children's immunity (Brame et al. 2024; Hanski et al. 2012; Roslund et al. 2020). Because the two Shannon–Weaver diversity indices did not meet the assumptions of normality and equality of variance, as assessed by the Shapiro–Wilk and Levene's tests, we used the Scheirer–Ray–Hare test to compare indices among vegetation and habitat types. All analyses were performed in R v. 4.5.2 software (R Core Team 2025).

## 3 Results

### 3.1 Data Characteristics

Classification identified 6146 unique ASVs in all 34 samples. The samples comprised 36 bacterial classes across 19 phyla with a relative abundance of > 1% (Figure 2). Pseudomonadota was a major phylum shared across the air, litter and soil samples. The other major phyla in the soil and litter samples were the Acidobacteriota, Actinobacteria and Planctomycetotia. The major classes in the soil and litter samples were Alphaproteobacteria, Gammaproteobacteria, Actinobacteria, Bacteroidia, Planctomycetacia and Verrucomicrobiia. The other major phyla in the air samples, on the other hand, were the Bacteroidota and Bacillota. The major classes in the air samples were Gammaproteobacteria, Bacteroidia and Bacilli. No clear differences in bacterial classes between vegetation type were evident.

**FIGURE 2:**
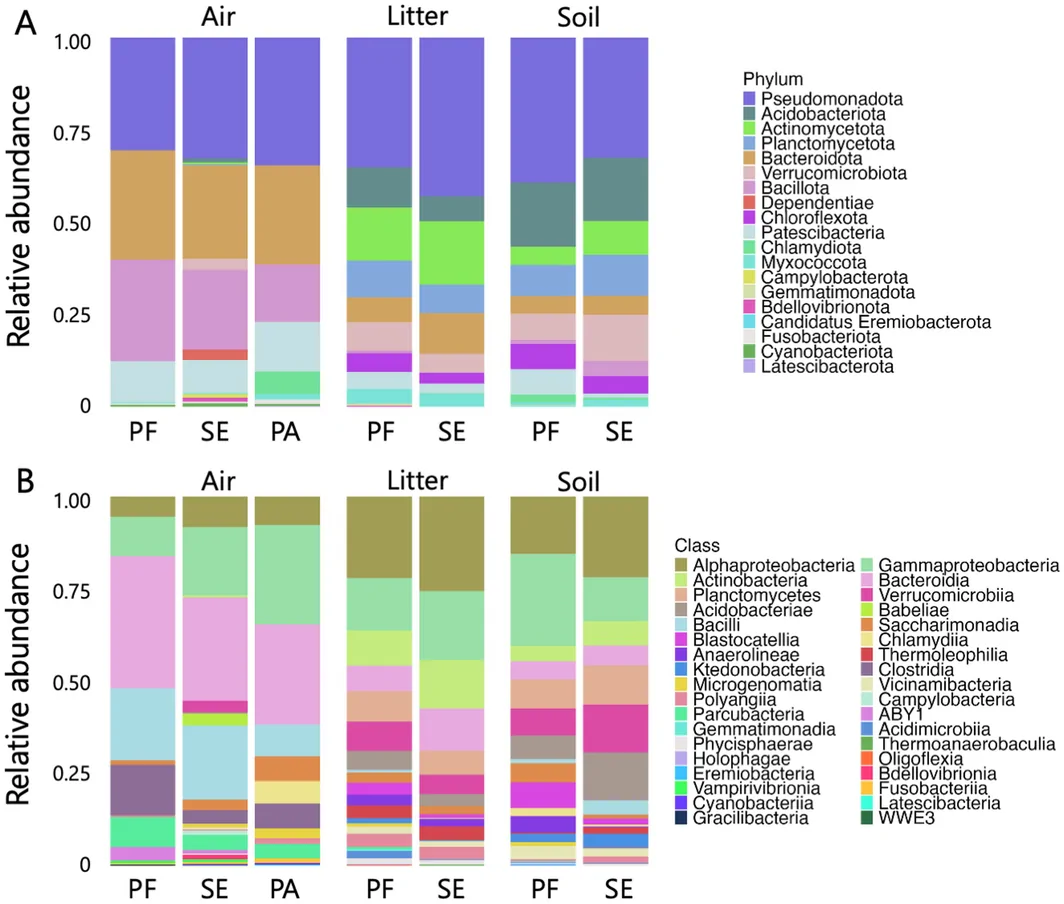
Relative abundance of bacterial taxa at the (A) phylum (left) and (B) class (right) levels. PA, parking area; PF, plantation forest; SF, secondary forest. The litter and soil samples from the parking area were excluded because they were collected from garden beds. We selected only those with a relative abundance of > 2% (see also Sections 2.2 and 2.4).

We found 95 genera in air, 354 in litter and 269 in soil (Figure 3A–C). The secondary forest had the highest number of bacterial genera in the air (Figure 3A). Over half of the bacterial genera in litter and soil were common across forest types (Figure 3B,C). In both forests, the highest number of genera was detected in litter (Figure 3D,E). Overall, more airborne bacterial genera were detected in the secondary forest than in the plantation forest (Figure 3D,E). Of the airborne genera, 39.7% were common with soil or litter in the secondary forest and 29.3% in the plantation forest. Similar patterns occurred at other taxonomic levels (Figures S2–S6).

**FIGURE 3:**
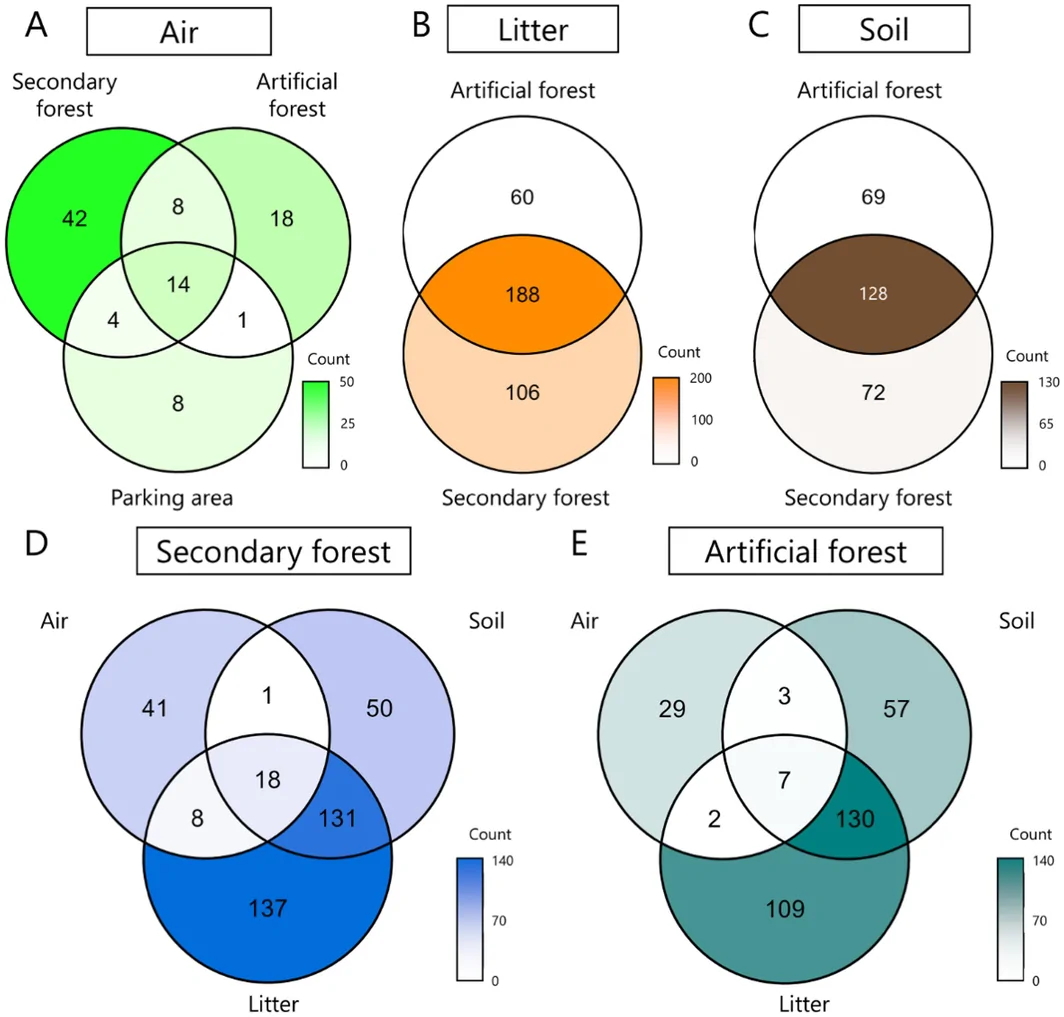
Number of common genera in vegetation types (A: Air, B: Litter, C: Surface soil) and habitats (D: Secondary forest, E: Plantation forest). A deeper colour indicates a greater number. Other taxonomic and ASV levels are summarized in Supplementary Figures.

We found 42 major genera (> 1% relative abundance in each habitat and vegetation type) in the samples (Figure 4). Those that were abundant in both soil and litter included *Bradyrhizobium*, *Candidatus* Udaeobacter and the *Burkholderia*–*Caballeronia*–*Paraburkholderia* complex. Those that were abundant in air, in contrast, were distinct from those in soil and litter and included *Flavobacterium* and *Enhydrobacter*. Minor genera (< 1% relative abundance) accounted for 40.8% of the litter communities in the secondary forest and 32.0% in the plantation forest; 22.8% in soil in the secondary forest and 14.3% in the plantation forest; and 13.9% in air in the secondary forest, 11.4% in the plantation forest and 5.7% in the parking area.

**FIGURE 4:**
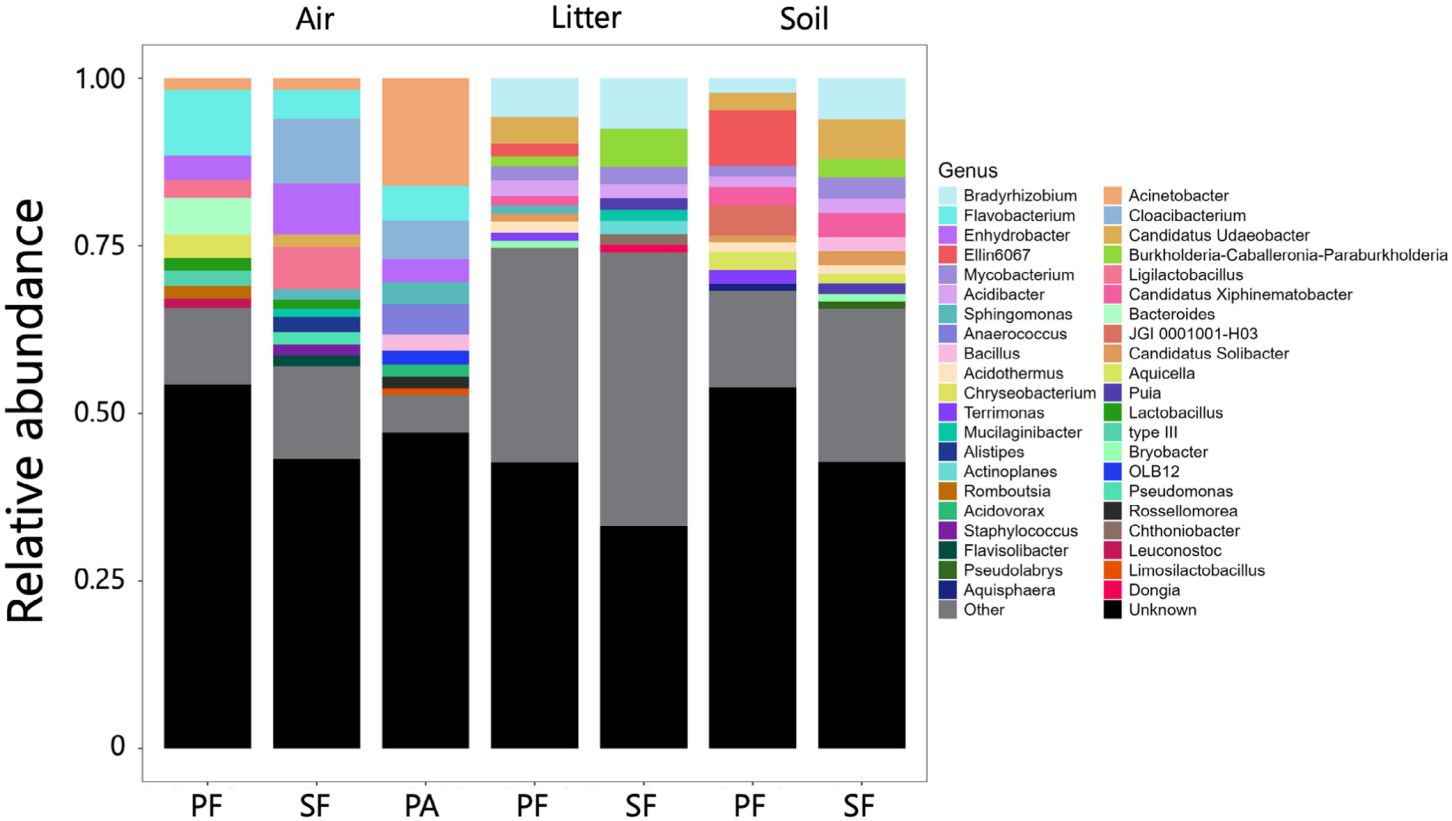
Proportions of dominant genera (> 1% relative abundance) among habitats and vegetation types. Genera with < 1% relative abundance are shown as ‘Other’. PA, parking area; PF, plantation forest; SF, secondary forest.

### 3.2 Microbial Community Structure and Diversity Among Habitats and Vegetation Types

The NMDS plot shows that the airborne samples were clearly separated from those in the ground habitats, indicating that the bacterial community structure differed across the habitats (Figure 5). PERMANOVA confirmed significant differences among the habitats (*p* < 0.001) and that the airborne bacterial community differed significantly from the soil and litter communities (both *p* < 0.001). The NMDS plot and PERMANOVA (*p* < 0.01) indicate that the bacterial community differed also across the vegetation types. PERMANOVA showed that the bacterial community in the plantation forest was significantly different from that in the secondary forest (*p* < 0.01).

**FIGURE 5:**
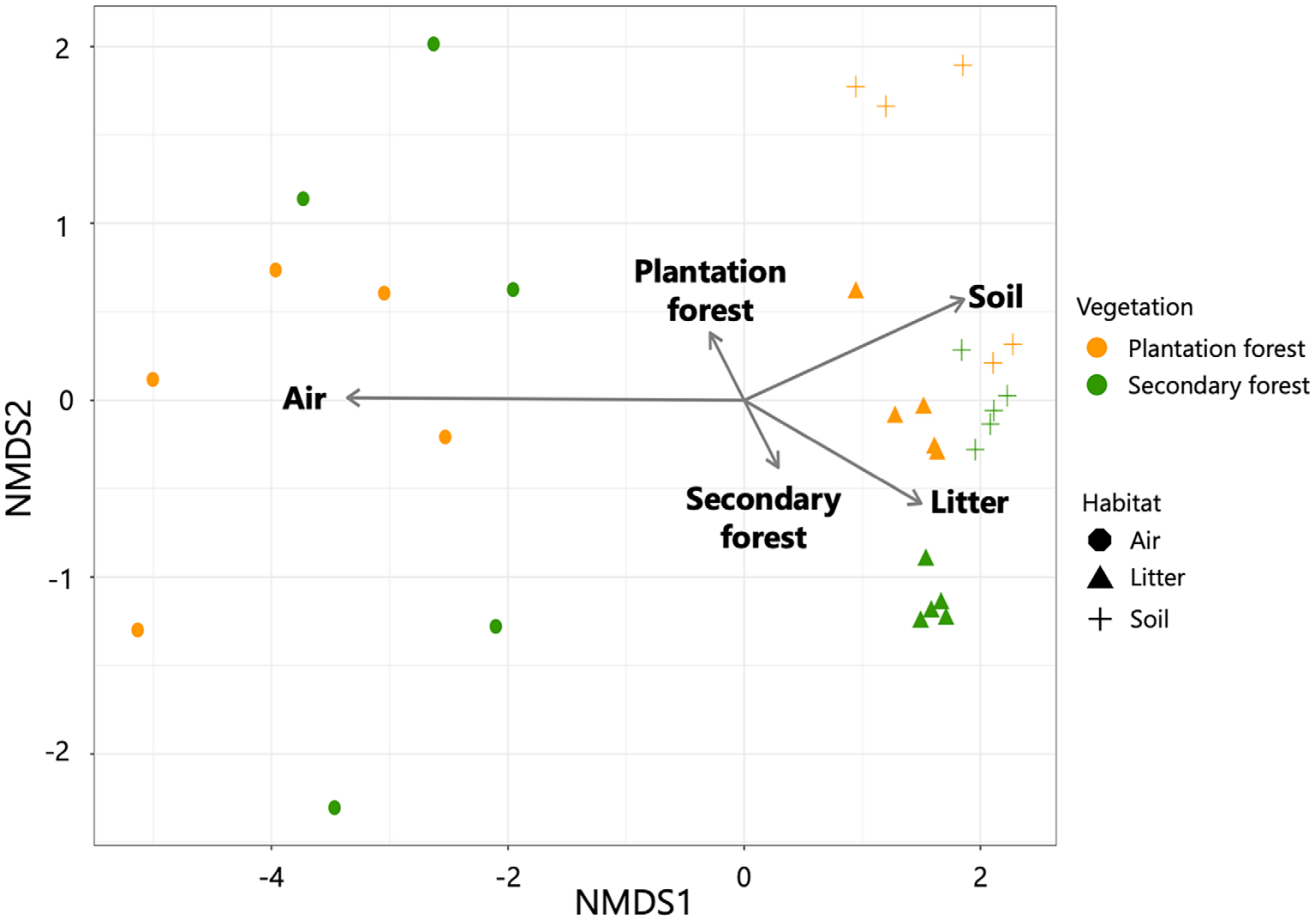
Plot of samples in nonmetric multidimensional scaling (NMDS) ordination space with an overlay of habitats and vegetation types. Each point represents a sample, each arrow an environmental variable. Stress value was 0.08, and goodness‐of‐fit in habitats was < 0.05.

The bacterial α diversity, as estimated by the Shannon–Weaver diversity index, differed significantly across the habitats (Figure 6 and Figure S7; *p* < 0.05, Scheirer–Ray–Hare test). That of the ground was higher than that of the air (Figure 6). The non‐parametric test, however, did not detect a significant difference between the vegetation types, although the small sample size limited statistical power.

**FIGURE 6:**
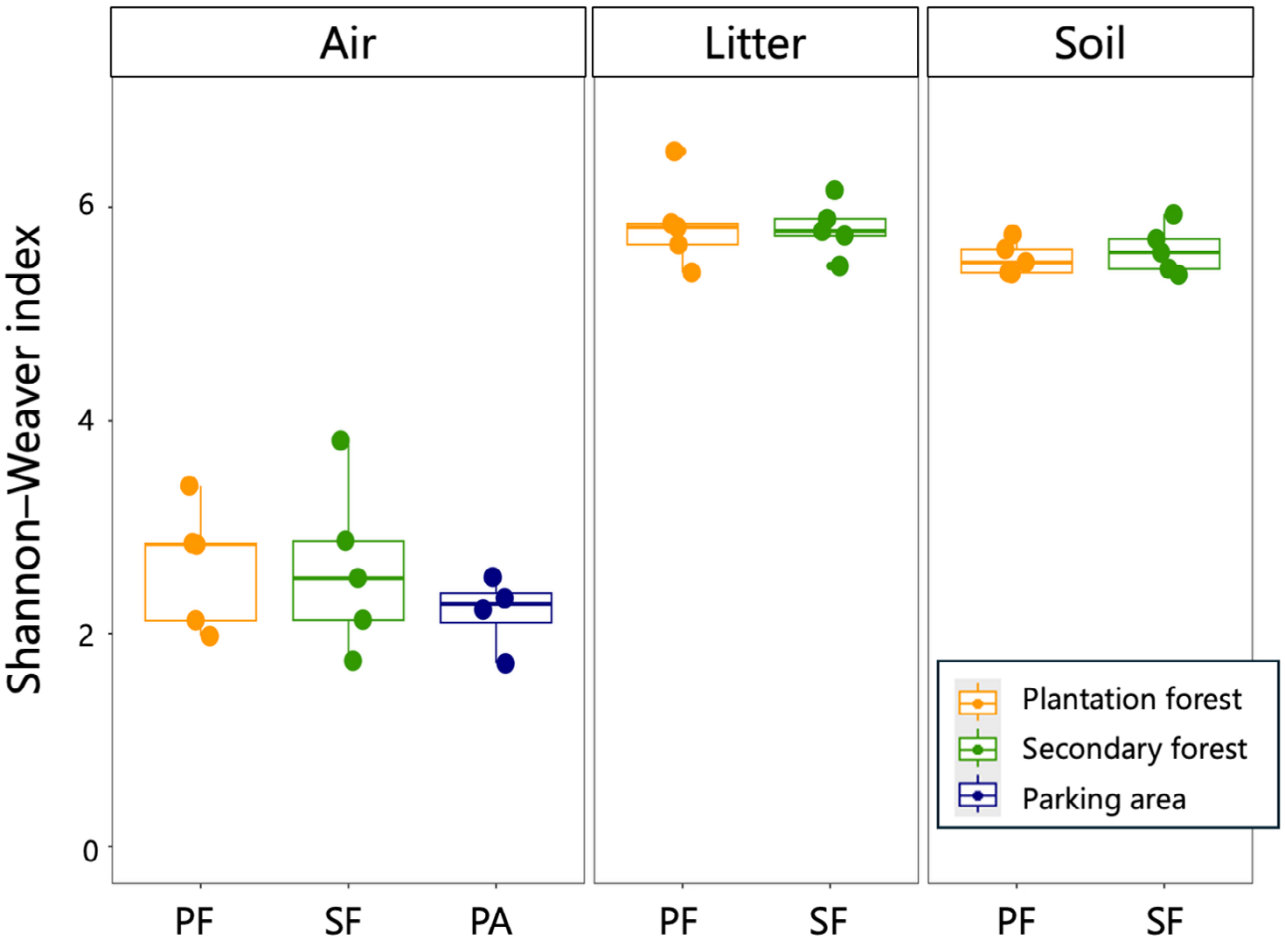
Bacterial α diversity, Shannon–Weaver index, of seven samples. PA, parking area; PF, plantation forest; SF, secondary forest.

## 4 Discussion

### 4.1 Differences in Bacterial Communities Among Air, Soil and Litter

Results showed differences in bacterial structure among air, soil and litter (Figures 2, 3, 4, 5). Although the airborne bacterial community was distinct from those of soil and litter (Figures 4 and 5), we found various common genera across air and ground (soil and litter) habitats (Figure 3), and the dominant taxonomic group was consistent among the three habitats (Figure 2). The results indicate that terrestrial sources contribute, at least partially, to airborne communities (Robinson et al. 2021), whereas less abundant taxa found only in aerosol samples may reflect atmospheric filtering that selects stress‐tolerant taxa, regardless of their source environments (Park and Fowler 2026). In addition, because 16S rRNA metabarcoding does not distinguish DNA derived from viable and nonviable cells, some taxa detected in air samples may represent aerosolized dead cells rather than actively dispersing bacterial populations (Park and Fowler 2026). For this reason, similarities among habitats should be interpreted with caution, although these observations remain consistent with the hypothesis that terrestrial habitats contribute to airborne bacterial DNA.

The Pseudomonadota accounted for a large proportion of the microbial communities in all habitats (Figure 2). While the Bacillota often become dominant in the atmosphere (Matulová et al. 2014; Walters et al. 2022) because of their tolerance to desiccation, UV radiation, heat and nutrient deprivation owing to protective mechanisms such as the spore coat (Nicholson et al. 2000; Setlow 2006), other phyla, including the Pseudomonadota, can account for a large proportion depending on airborne habitat conditions (Vaïtilingom et al. 2012). In addition to possible contributions from taxa shared with litter and soil (Figure 3), the large proportion of Pseudomonadota in airborne bacterial communities might also be influenced by the release of bacteria from leaf surfaces (Lymperopoulou et al. 2016). A more comprehensive analysis of forest environmental bacterial communities, including those associated with plant surfaces, is an important direction for future research. Gammaproteobacteria, whose environmental and skin‐associated diversity has been associated with human immune regulation in previous studies (Hanski et al. 2012; Roslund et al. 2020), were consistently present in air, litter and soil, although they were not dominant in forest air (Figure 2). Combined with previous findings that airborne bacteria may have only a limited influence on the human microbiome (Brame et al. 2024), this pattern indicates that the link between airborne bacterial exposure and the human immune system may not necessarily be strong.

Despite the common phyla and classes, the NMDS plot and statistical tests show a significant difference in the bacterial communities among the habitats at the ASV level (Figure 5) and in the dominant bacterial groups between the air and ground habitats at the genus level (Figure 4). These results indicate that even among bacteria in the same phylum or class, taxa that are well adapted to each environment differ among air, soil and litter at the genus and species levels. Airborne bacteria had a different structure from ground bacteria (Figure 5) and lower biodiversity than in soil and litter (Figure 6). These results can be partly explained by the harshness of the aerial environment, which can limit the taxonomic diversity of bacteria that can be detected through the 16S rRNA metabarcoding (Archer et al. 2023). In addition, lower‐abundance taxa often cause bacterial community differences (Mhuireach et al. 2021). The airborne bacterial structure might be formed by unique, rare taxa screened out by the environmental filter. It should be noted that airborne pathogens including bacteria can move long distances (Šantl‐Temkiv et al. 2022) although the impact of bacterial dispersal on community structure depends on its pathway and the source habitat (Barbour et al. 2023; Walters et al. 2022). A larger variation in the airborne bacteria community than in the ground ones (Figure 5) might reflect the mobility of the airborne bacteria.

### 4.2 Differences in Bacterial Communities Among Vegetation Types

Our survey also showed differences in bacterial structure between plantation and secondary forests at the ASV level (Figure 5), as previously reported in Japan (Nakayama et al. 2019). This result is consistent with reports that the composition of airborne bacterial communities varies with vegetation (Mhuireach et al. 2021; Robinson et al. 2021). The main tree species composition of forest can influence the surrounding chemical environment (Urbanová et al. 2015), thus potentially affecting the microbial community therein (Nakayama et al. 2019). The plantation forest at our site is composed entirely of *C. obtusa* trees, whereas the secondary one is composed of *Q. serrata* , *Q. acutissima* and *C. jamasakura* trees, along with diverse understory vegetation. Such diversity may contribute to differences in bacterial structure, potentially due to variations in the nutrient availability between the two forest types through litter‐derived or root‐derived pathways, which modify the soil nutrient environment (Nakayama et al. 2019). Furthermore, approximately half of the genera were unique in each vegetation type in soil and litter (Figure 3B,C), but most of the unique ones were not dominant (Figure 4). The bacterial diversity was also not significantly different among the vegetation types (Figure 6). These results suggest that nutrient differences arising from vegetation (Xue et al. 2017) determine the composition of non‐dominant bacteria and the community structure, but do not cause differences in bacterial diversity (Hanski et al. 2012). Further research is needed to determine which pathway influences the ground and airborne bacterial structures.

### 4.3 Relationship Between Bacterial Diversity in the Forest Ecosystem and Human Well‐Being

We found fewer bacterial genera in the air than in the ground (Figure 3A–C), with lower biodiversity (Figure 6). Nevertheless, several genera overlapped between the air and ground habitats (Figure 3). Shared genera within the Alphaproteobacteria (e.g., *Sphingomonas*) and the Gammaproteobacteria (e.g., *Pseudomonas*) have been linked to a human immune system (Hanski et al. 2012; Roslund et al. 2020). Furthermore, unique genera were present in the air in both forests, at 10.6% in the secondary forest and 8.6% in the plantation forest (Figure 3D,E). Most of these unique bacteria belong to rare taxa, apart from *Enhydrobacter*, *Flavobacterium* and *Ligilactobacillus* (Figure 4).

### 4.4 Limitations

Several limitations should be acknowledged. Notably, there were fewer DNA reads in the air samples than in the litter and soil samples (Figure 3) on account of the much lower bacterial biomass in the air than in litter and soil (Baldrian 2016). Although ground‐based air sampling methods have been compared (Kalisa et al. 2022), they are still under discussion. In urban areas, a high‐volume sampler (1000 L/min) is often used to collect aerosols (Hu et al. 2020; Kalisa et al. 2022). At our site, however, it was too difficult for us to use these because of the steep slope, the lack of a power supply and the risk of rain. Although we ran our samplers for a week to filter > 10,000 L of air, the low flow rate (1.5 L/min) may have undersampled rare taxa, affecting the diversity estimates (see Tables S1 and S2 in the Supplementary Information for the report of DNA yields and sequencing depths). To increase DNA yield from air samples, filtration methods may be as important as those for water samples (Hinlo et al. 2017), but filter materials and pore sizes are not standardized; two studies used glass fibre filters (Hu et al. 2020; Kalisa et al. 2022), and one used polycarbonate filters of 0.22 μm (Maki et al. 2023). Comparisons of flow rates and filters are necessary to find better ground‐based air sampling methods. In addition, future studies may benefit from integrating 16S rRNA metabarcoding with complementary approaches to provide deeper insights into microbial identity, distribution and abundance. Furthermore, although this study focused on a single summer sampling period when the forest campsite is frequently used by visitors, examining seasonal variation in airborne, litter and soil bacterial communities would be valuable, as their relationships may change with meteorological conditions and plant phenology.

## 5 Conclusion

Our study at a forest campsite, based on sampling more than 10,000 L of air per device over 7 days, showed that airborne bacterial communities partially overlapped with those in litter and soil at the ASV and genus levels. This overlap suggests contributions from terrestrial sources, whereas less abundant taxa detected only in airborne samples may reflect atmospheric filtering that favours stress‐tolerant taxa. By separately sampling the litter layer, a critical yet understudied interface between the atmosphere and soil, we revealed taxonomic overlap and differentiation not only between soil and air but also between litter and air. This pattern was consistent in both plantation and secondary forests, which differed in plant diversity and human use. The structure of the bacterial community could also be influenced by vegetation type, likely in relation to vegetation diversity and nutrient availability. These results contribute to understanding both shared and habitat‐specific components of bacterial communities in the air, litter and soil of forests and the relationship between exposure to the environmental microbiome and human well‐being.

## Author Contributions

**Keiichi Kimura:** conceptualization, data curation, formal analysis, investigation, methodology, visualization, writing – original draft, writing – review and editing. **Natsuko I. Kondo:** conceptualization, data curation, formal analysis, investigation, methodology, writing – review and editing. **Nobuyoshi Nakajima:** methodology, writing – review and editing. **Hiroshi C. Ito:** methodology, writing – review and editing. **Taku Kadoya:** conceptualization, writing – review and editing. **Kazuaki Tsuchiya:** conceptualization, data curation, funding acquisition, investigation, project administration, supervision, writing – review and editing.

## Funding

This work was supported by the Environmental Restoration and Conservation Agency (JPMEERF20241006).

## Ethics Statement

The authors have nothing to report.

## Conflicts of Interest

The authors declare no conflicts of interest.

## Supporting information


**Supplementary Figure 1.** Rarefaction curves for each sample. The red dot line shows the minimum reads (EC008), 9544 sequences. We rarefied each sample to this minimum sequence for downstream analyses.
**Supplementary Figure 2.** Number of common phyla in vegetation types (upper) and habitats (lower).
**Supplementary Figure 3.** Number of common classes in vegetation types (upper) and habitats (lower).
**Supplementary Figure 4.** Number of common orders in vegetation types (upper) and habitats (lower).
**Supplementary Figure 5.** Number of common families in vegetation types (upper) and habitats (lower).
**Supplementary Figure 6.** Number of common amplicon sequence variants in vegetation types (upper) and habitats (lower).
**Supplementary Figure 7.** Bacterial α diversity, Shannon–Weaver index, of the Gammaproteobacteria class. PA, parking area; PF, plantation forest; SF, secondary forest.
**Table S1:** DNA yields from air, litter and soil samples measured using the TapeStation. The detection limit for genomic DNA was 0.5 ng/μL. nd, not detected (below the detection limit).
**Table S2:** Sequencing depths (numbers of sequences) for air, litter and soil samples at each stage of sequence processing. Columns are arranged from left to right according to the workflow from raw reads to the sequences retained for the final analysis. Abbreviations: rawF, raw forward reads; rawR, raw reverse reads; nopriF, forward reads after primer removal; nopriR, reverse reads after primer removal; filtF, filtered forward reads; filtR, filtered reverse reads; denF, denoised forward reads; denR, denoised reverse reads; merged, merged forward and reverse reads; len_filt, reads retained after length filtering; and nochmr, reads retained after chimera removal. During length filtering, sequences of 240–320 bp were retained. Further details of sequence processing are provided in Section 2.3.

## Data Availability

The sequence data have been deposited in the DDBJ Sequence Read Archive under accession numbers DRR1053300‐DRR1053333 (Bioproject: PRJDB42365). Data and the R code used for analyses are available from the corresponding author on reasonable request.

## References

[emi470427-bib-0001] Aota, Y. , K. Tsuchiya , S. Endo , and M. Soga . 2025. “High Vulnerability of Children's Wildlife‐Oriented Outdoor Activities to Urbanization and Digital Media Use.” Landscape and Urban Planning 262: 105408. 10.1016/j.landurbplan.2025.105408.

[emi470427-bib-0002] Archer, S. D. J. , K. C. Lee , T. Caruso , et al. 2023. “Contribution of Soil Bacteria to the Atmosphere Across Biomes.” Science of the Total Environment 871: 162137. 10.1016/j.scitotenv.2023.162137. 36775167

[emi470427-bib-0003] Baldrian, P. 2016. “Forest Microbiome: Diversity, Complexity and Dynamics.” FEMS Microbiology Reviews fuw040: fuw040. 10.1093/femsre/fuw040. 27856492

[emi470427-bib-0004] Barbour, K. M. , A. Barrón‐Sandoval , K. E. Walters , and J. B. H. Martiny . 2023. “Towards Quantifying Microbial Dispersal in the Environment.” Environmental Microbiology 25, no. 1: 137–142. 10.1111/1462-2920.16270. PMC10100412 36308707

[emi470427-bib-0005] Brame, J. E. , I. Warbrick , D. Heke , C. Liddicoat , and M. F. Breed . 2024. “Short‐Term Passive Greenspace Exposures Have Little Effect on Nasal Microbiomes: A Cross‐Over Exposure Study of a Māori Cohort.” Environmental Research 252: 118814. 10.1016/j.envres.2024.118814. 38555095

[emi470427-bib-0006] Callahan, B. 2024. “Silva Taxonomic Training Data Formatted for DADA2 (Silva Version 138.2) [Data set].” Zenodo. 10.5281/zenodo.14169026.

[emi470427-bib-0007] Callahan, B. J. , P. J. McMurdie , M. J. Rosen , A. W. Han , A. J. A. Johnson , and S. P. Holmes . 2016. “DADA2: High‐Resolution Sample Inference From Illumina Amplicon Data.” Nature Methods 13: 581–583. 10.1038/nmeth.3869. PMC4927377 27214047

[emi470427-bib-0008] Caporaso, J. G. , C. L. Lauber , W. A. Walters , et al. 2011. “Global Patterns of 16S rRNA Diversity at a Depth of Millions of Sequences Per Sample.” Proceedings of the National Academy of Sciences 108: 4516–4522. 10.1073/pnas.1000080107. PMC3063599 20534432

[emi470427-bib-0009] Davis, N. M. , D. M. Proctor , S. P. Holmes , D. A. Relman , and B. J. Callahan . 2018. “Simple Statistical Identification and Removal of Contaminant Sequences in Marker‐Gene and Metagenomics Data.” Microbiome 6: 226. 10.1186/s40168-018-0605-2. PMC6298009 30558668

[emi470427-bib-0010] Haahtela, T. 2019. “A Biodiversity Hypothesis.” Allergy 74, no. 8: 1445–1456. 10.1111/all.13763. 30835837

[emi470427-bib-0011] Hanski, I. , L. von Hertzen , N. Fyhrquist , et al. 2012. “Environmental Biodiversity, Human Microbiota, and Allergy Are Interrelated.” Proceedings of the National Academy of Sciences 109: 8334–8339. 10.1073/pnas.1205624109. PMC3361383 22566627

[emi470427-bib-0012] Heederik, D. , and E. Von Mutius . 2012. “Does Diversity of Environmental Microbial Exposure Matter for the Occurrence of Allergy and Asthma?” Journal of Allergy and Clinical Immunology 130: 44–50. 10.1016/J.JACI.2012.01.067. 22502794

[emi470427-bib-0013] Hinlo, R. , D. Gleeson , M. Lintermans , and E. Furlan . 2017. “Methods to Maximise Recovery of Environmental DNA From Water Samples.” PLoS One 12: e0179251. 10.1371/journal.pone.0179251. PMC5467897 28604830

[emi470427-bib-0014] Hu, Z. , H. Liu , H. Zhang , et al. 2020. “Temporal Discrepancy of Airborne Total Bacteria and Pathogenic Bacteria Between Day and Night.” Environmental Research 186: 109540. 10.1016/J.ENVRES.2020.109540. 32339956

[emi470427-bib-0015] Isabwe, A. , K. Ren , Y. Wang , F. Peng , H. Chen , and J. Yang . 2019. “Community Assembly Mechanisms Underlying the Core and Random Bacterioplankton and Microeukaryotes in a River–Reservoir System.” Water 11, no. 6: 1127. 10.3390/w11061127.

[emi470427-bib-0016] Kalisa, E. , V. Kuuire , and M. Adams . 2022. “A Preliminary Investigation Comparing High‐Volume and Low‐Volume Air Samplers for Measurement of PAHs, NPAHs and Airborne Bacterial Communities in Atmospheric Particulate Matter.” Environmental Science: Atmospheres 2: 1120–1131. 10.1039/D2EA00078D.

[emi470427-bib-0017] Lashus, D. C. , A. Gomez , T. Hummel , et al. 2026. “Associations of Forest vs. Urban Environmental Exposure With Well‐Being and Nasal Microbiome Composition: An Exploratory Pilot Study.” Environmental Research 291: 123582. 10.1016/j.envres.2025.123582. PMC12950270 41443494

[emi470427-bib-0018] Lymperopoulou, D. S. , R. I. Adams , and S. E. Lindow . 2016. “Contribution of Vegetation to the Microbial Composition of Nearby Outdoor Air.” Applied and Environmental Microbiology 82, no. 13: 3822–3833. 10.1128/AEM.00610-16. PMC4907200 27107117

[emi470427-bib-0019] Maki, T. , K. Hosaka , K. C. Lee , et al. 2023. “Vertical Distribution of Airborne Microorganisms Over Forest Environments: A Potential Source of Ice‐Nucleating Bioaerosols.” Atmospheric Environment 302: 119726. 10.1016/J.ATMOSENV.2023.119726.

[emi470427-bib-0020] Martin, M. 2011. “Cutadapt Removes Adapter Sequences From High‐Throughput Sequencing Reads.” EMBnet. Journal 17: 10. 10.14806/ej.17.1.200.

[emi470427-bib-0021] Matulová, M. , S. Husárová , P. Capek , M. Sancelme , and A.‐M. Delort . 2014. “Biotransformation of Various Saccharides and Production of Exopolymeric Substances by Cloud‐Borne *Bacillus* sp. 3B6.” Environmental Science & Technology 48: 14238–14247. 10.1021/es501350s. 25390890

[emi470427-bib-0022] Mhuireach, G. , B. R. Johnson , A. E. Altrichter , et al. 2016. “Urban Greenness Influences Airborne Bacterial Community Composition.” Science of the Total Environment 571: 680–687. 10.1016/J.SCITOTENV.2016.07.037. 27418518

[emi470427-bib-0023] Mhuireach, G. Á. , H. Wilson , and B. R. Johnson . 2021. “Urban Aerobiomes Are Influenced by Season, Vegetation, and Individual Site Characteristics.” EcoHealth 18: 331–344. 10.1007/s10393-020-01493-w. 33170406

[emi470427-bib-0024] Ministry of the Environment of Japan . 2025. “Japan's OECM and Related Policy.” https://www.env.go.jp/nature/biodiversity/OECM.html.

[emi470427-bib-0025] Nakayama, M. , S. Imamura , T. Taniguchi , and R. Tateno . 2019. “Does Conversion From Natural Forest to Plantation Affect Fungal and Bacterial Biodiversity, Community Structure, and Co‐Occurrence Networks in the Organic Horizon and Mineral Soil?” Forest Ecology and Management 446: 238–250. 10.1016/j.foreco.2019.05.042.

[emi470427-bib-0026] Nearing, J. T. , G. M. Douglas , A. M. Comeau , and M. G. I. Langille . 2018. “Denoising the Denoisers: An Independent Evaluation of Microbiome Sequence Error‐Correction Approaches.” PeerJ 6: e5364. 10.7717/peerj.5364. PMC6087418 30123705

[emi470427-bib-0027] Nicholson, W. L. , N. Munakata , G. Horneck , H. J. Melosh , and P. Setlow . 2000. “Resistance of Bacillus Endospores to Extreme Terrestrial and Extraterrestrial Environments.” Microbiology and Molecular Biology Reviews 64, no. 3: 548–572. 10.1128/mmbr.64.3.548-572.2000. PMC99004 10974126

[emi470427-bib-0028] Park, J. , and S. J. Fowler . 2026. “Airborne Bacterial Communities: Diversity, Survival Strategies and Functional Roles in the Atmosphere.” Environmental Microbiology Reports 18, no. 1: e70274. 10.1111/1758-2229.70274. PMC12784115 41508819

[emi470427-bib-0029] R Core Team . 2025. “R: A Language and Environment for Statistical Computing.”

[emi470427-bib-0030] Robinson, J. M. , and M. F. Breed . 2023. “The Aerobiome–Health Axis: A Paradigm Shift in Bioaerosol Thinking.” Trends in Microbiology 31: 661–664. 10.1016/j.tim.2023.04.007. 37211511

[emi470427-bib-0031] Robinson, J. M. , C. Cando‐Dumancela , R. E. Antwis , et al. 2021. “Exposure to Airborne Bacteria Depends Upon Vertical Stratification and Vegetation Complexity.” Scientific Reports 11: 9516. 10.1038/s41598-021-89065-y. PMC8096821 33947905

[emi470427-bib-0032] Robinson, J. M. , C. Cando‐Dumancela , C. Liddicoat , P. Weinstein , R. Cameron , and M. F. Breed . 2020. “Vertical Stratification in Urban Green Space Aerobiomes.” Environmental Health Perspectives 128: 117008. 10.1289/EHP7807. PMC7687659 33236934

[emi470427-bib-0033] Roslund, M. I. , R. Puhakka , M. Grönroos , et al. 2020. “Biodiversity Intervention Enhances Immune Regulation and Health‐Associated Commensal Microbiota Among Daycare Children.” Science Advances 6: eaba2578. 10.1126/sciadv.aba2578. PMC7556828 33055153

[emi470427-bib-0034] Šantl‐Temkiv, T. , P. Amato , E. O. Casamayor , P. K. Lee , and S. B. Pointing . 2022. “Microbial Ecology of the Atmosphere.” FEMS Microbiology Reviews 46, no. 4: fuac009. 10.1093/femsre/fuac009 PMC9249623 35137064

[emi470427-bib-0035] Šantl‐Temkiv, T. , P. Amato , U. Gosewinkel , et al. 2017. “High‐Flow‐Rate Impinger for the Study of Concentration, Viability, Metabolic Activity, and Ice‐Nucleation Activity of Airborne Bacteria.” Environmental Science & Technology 51, no. 19: 11224–11234. 10.1021/acs.est.7b01480. 28836763

[emi470427-bib-0036] Selway, C. A. , J. G. Mills , P. Weinstein , et al. 2020. “Transfer of Environmental Microbes to the Skin and Respiratory Tract of Humans After Urban Green Space Exposure.” Environment International 145: 106084. 10.1016/J.ENVINT.2020.106084. 32977191

[emi470427-bib-0037] Setlow, P. 2006. “Spores of *Bacillus subtilis* : Their Resistance to and Killing by Radiation, Heat and Chemicals.” Journal of Applied Microbiology 101, no. 3: 514–525. 10.1111/j.1365-2672.2005.02736.x. 16907802

[emi470427-bib-0038] Smets, W. , S. Moretti , S. Denys , and S. Lebeer . 2016. “Airborne Bacteria in the Atmosphere: Presence, Purpose, and Potential.” Atmospheric Environment 139: 214–221. 10.1016/J.ATMOSENV.2016.05.038.

[emi470427-bib-0039] Urbanová, M. , J. Šnajdr , and P. Baldrian . 2015. “Composition of Fungal and Bacterial Communities in Forest Litter and Soil Is Largely Determined by Dominant Trees.” Soil Biology and Biochemistry 84: 53–64. 10.1016/j.soilbio.2015.02.011.

[emi470427-bib-0040] Vaïtilingom, M. , E. Attard , N. Gaiani , et al. 2012. “Long‐Term Features of Cloud Microbiology at the Puy de Dôme (France).” Atmospheric Environment 56: 88–100. 10.1016/j.atmosenv.2012.03.072.

[emi470427-bib-0041] Walters, K. E. , J. K. Capocchi , M. B. N. Albright , Z. Hao , E. L. Brodie , and J. B. H. Martiny . 2022. “Routes and Rates of Bacterial Dispersal Impact Surface Soil Microbiome Composition and Functioning.” ISME Journal 16: 2295–2304. 10.1038/s41396-022-01269-w. PMC9477824 35778440

[emi470427-bib-0042] Xue, L. , H. Ren , S. Li , X. Leng , and X. Yao . 2017. “Soil Bacterial Community Structure and Co‐Occurrence Pattern During Vegetation Restoration in Karst Rocky Desertification Area.” Frontiers in Microbiology 8, no. 2017: 2377. 10.3389/fmicb.2017.02377 PMC5717032 29250053

