# Supplementary material for: Airborne Bacteria Are Influenced by Surrounding Soil, Litter and Vegetation at a Forest Campsite
Source: Environ Microbiol Rep. 2026 Oct 7;18(5):e70427. doi: 10.1111/1758-2229.70427 (PMC13643372; doi:10.1111/1758-2229.70427)
Supplement: Supplementary file 1 [file EMI4-18-e70427-s001.docx]

Supplementary Information

Airborne bacteria are influenced by surrounding soil, litter, and vegetation at a forest campsite

Keiichi Kimura^a^, Natsuko I. Kondo^b^, Nobuyoshi Nakajima^b^, Hiroshi C. Ito^b^, Taku Kadoya^b^, Kazuaki Tsuchiya^a^

^a^ Social Systems Division, National Institute for Environmental Studies, 16-2, Onogawa Tsukuba, Ibaraki, Japan

^b^ Biodiversity Division, National Institute for Environmental Studies, 16-2, Onogawa Tsukuba, Ibaraki, Japan

Supplementary Figure 1. Rarefaction curves for each sample. The red dot line shows the minimum reads (EC008), 9544 sequences. We rarefied each sample to this minimum sequence for downstream analyses.

Supplementary Figure 2. Number of common phyla in vegetation types (upper) and habitats (lower).

Supplementary Figure 3. Number of common classes in vegetation types (upper) and habitats (lower).

Supplementary Figure 4. Number of common orders in vegetation types (upper) and habitats (lower).

Supplementary Figure 5. Number of common families in vegetation types (upper) and habitats (lower).

Supplementary Figure 6. Number of common amplicon sequence variants in vegetation types (upper) and habitats (lower).

Supplementary Figure 7. Bacterial α diversity, Shannon–Weaver index, of the Gammaproteobacteria class. SF, secondary forest; PF, plantation forest; PA, parking area.

Table S1. DNA yields from air, litter and soil samples measured using the TapeStation. The detection limit for genomic DNA was 0.5 ng/μL. nd, not detected (below the detection limit).

Table S2. Sequencing depths (numbers of sequences) for air, litter, and soil samples at each stage of sequence processing. Columns are arranged from left to right according to the workflow from raw reads to the sequences retained for the final analysis. Abbreviations: rawF, raw forward reads; rawR, raw reverse reads; nopriF, forward reads after primer removal; nopriR, reverse reads after primer removal; filtF, filtered forward reads; filtR, filtered reverse reads; denF, denoised forward reads; denR, denoised reverse reads; merged, merged forward and reverse reads; len_filt, reads retained after length filtering; and nochmr, reads retained after chimera removal. During length filtering, sequences of 240–320 bp were retained. Further details of sequence processing are provided in Section 2.3.

Supplementary Figure 1. Rarefaction curves for each sample. The red dot line shows the minimum reads (EC008), 9544 sequences. We rarefied each sample to this minimum sequence for downstream analyses.

Supplementary Figure 2. Number of common phyla in vegetation types (upper) and habitats (lower).

Supplementary Figure 3. Number of common classes in vegetation types (upper) and habitats (lower).

Supplementary Figure 4. Number of common orders in vegetation types (upper) and habitats (lower).

Supplementary Figure 5. Number of common families in vegetation types (upper) and habitats (lower).

Supplementary Figure 6. Number of common amplicon sequence variants in vegetation types (upper) and habitats (lower).

Supplementary Figure 7. Bacterial α diversity, Shannon–Weaver index, of the Gammaproteobacteria class. SF, secondary forest; PF, plantation forest; PA, parking area.

Supplementary Table S1. DNA yields from air, litter, and soil samples measured using the TapeStation. The detection limit for genomic DNA was 0.5 ng/µL. nd, not detected (below the detection limit).

| Sampe ID | Habitat | Vegetation | TapeStation (ng/µL) |
| --- | --- | --- | --- |
| EC003 | air | Secondary forest | nd |
| EC004 | air | Secondary forest | nd |
| EC005 | air | Secondary forest | nd |
| EC006 | air | Secondary forest | nd |
| EC007 | air | Secondary forest | nd |
| EC008 | air | Plantation forest | nd |
| EC009 | air | Plantation forest | nd |
| EC010 | air | Plantation forest | nd |
| EC011 | air | Plantation forest | nd |
| EC012 | air | Plantation forest | nd |
| EC013 | air | Parking | nd |
| EC014 | air | Parking | nd |
| EC016 | air | Parking | nd |
| EC017 | air | Parking | nd |
| EC022 | soil | Secondary forest | 122.1 |
| EC023 | soil | Secondary forest | 216.6 |
| EC024 | soil | Secondary forest | 6.57 |
| EC025 | soil | Secondary forest | 1.01 |
| EC026 | soil | Secondary forest | 7.28 |
| EC027 | soil | Plantation forest | 62.1 |
| EC028 | soil | Plantation forest | 164.8 |
| EC029 | soil | Plantation forest | 0.865 |
| EC030 | soil | Plantation forest | 0.91 |
| EC031 | soil | Plantation forest | 0.758 |
| EC037 | litter | Secondary forest | 132 |
| EC038 | litter | Secondary forest | 211.2 |
| EC039 | litter | Secondary forest | 215.7 |
| EC040 | litter | Secondary forest | 191.1 |
| EC041 | litter | Secondary forest | 198.3 |
| EC042 | litter | Plantation forest | 209.7 |
| EC043 | litter | Plantation forest | 206.7 |
| EC044 | litter | Plantation forest | 0.859 |
| EC045 | litter | Plantation forest | 189.9 |
| EC046 | litter | Plantation forest | 0.808 |

Supplementary Table S2. Sequencing depths (numbers of sequences) for air, litter, and soil samples at each stage of sequence processing. Columns are arranged from left to right according to the workflow from raw reads to the sequences retained for the final analysis. Abbreviations: rawF, raw forward reads; rawR, raw reverse reads; nopriF, forward reads after primer removal; nopriR, reverse reads after primer removal; filtF, filtered forward reads; filtR, filtered reverse reads; denF, denoised forward reads; denR, denoised reverse reads; merged, merged forward and reverse reads; len_filt, reads retained after length filtering; and nochmr, reads retained after chimera removal. During length filtering, sequences of 240–320 bp were retained. Further details of sequence processing are provided in Section 2.3.

| ID | habitat | environment | rawF | rawR | nopriF | nopriR | filtF | filtR | denF | denR | merged | len_filt | nochmr |
| --- | --- | --- | --- | --- | --- | --- | --- | --- | --- | --- | --- | --- | --- |
| EC003 | air | Secondary forest | 61679 | 61679 | 61679 | 61679 | 61624 | 61624 | 60035 | 60412 | 54278 | 31560 | 25730 |
| EC004 | air | Secondary forest | 183235 | 183235 | 183235 | 183235 | 182276 | 182276 | 181681 | 181195 | 164947 | 15941 | 11484 |
| EC005 | air | Secondary forest | 44667 | 44667 | 44667 | 44667 | 44576 | 44576 | 43509 | 43640 | 38004 | 19429 | 14380 |
| EC006 | air | Secondary forest | 195290 | 195290 | 195290 | 195290 | 194978 | 194978 | 188300 | 187611 | 179221 | 85144 | 51573 |
| EC007 | air | Secondary forest | 60410 | 60410 | 60410 | 60410 | 60354 | 60354 | 58409 | 57869 | 54945 | 28590 | 23122 |
| EC008 | air | Plantation forest | 49394 | 49394 | 49394 | 49394 | 49229 | 49229 | 48019 | 46708 | 29662 | 15094 | 9544 |
| EC009 | air | Plantation forest | 51636 | 51636 | 51636 | 51636 | 51521 | 51521 | 50344 | 50529 | 46270 | 17493 | 13815 |
| EC010 | air | Plantation forest | 101884 | 101884 | 101884 | 101884 | 101775 | 101775 | 98729 | 98997 | 76805 | 35105 | 24046 |
| EC011 | air | Plantation forest | 63464 | 63464 | 63464 | 63464 | 63423 | 63423 | 61186 | 61235 | 56546 | 29499 | 22153 |
| EC012 | air | Plantation forest | 55115 | 55115 | 55115 | 55115 | 55037 | 55037 | 53716 | 54055 | 49819 | 24298 | 17898 |
| EC013 | air | Parking | 38355 | 38355 | 38355 | 38355 | 38237 | 38237 | 36568 | 36350 | 34310 | 17788 | 12931 |
| EC014 | air | Parking | 37914 | 37914 | 37914 | 37914 | 37898 | 37898 | 37076 | 36605 | 34319 | 29758 | 23807 |
| EC016 | air | Parking | 34311 | 34311 | 34311 | 34311 | 34239 | 34239 | 32541 | 32230 | 27210 | 16368 | 12425 |
| EC017 | air | Parking | 58323 | 58323 | 58323 | 58323 | 58196 | 58196 | 56316 | 55345 | 53151 | 34254 | 26039 |
| EC022 | soil | Secondary forest | 127656 | 127656 | 127656 | 127656 | 127629 | 127629 | 109352 | 106969 | 69112 | 68896 | 67729 |
| EC023 | soil | Secondary forest | 104307 | 104307 | 104307 | 104307 | 104297 | 104297 | 90510 | 88200 | 60380 | 60153 | 59316 |
| EC024 | soil | Secondary forest | 101900 | 101900 | 101900 | 101900 | 101878 | 101878 | 85652 | 82285 | 54422 | 53546 | 51980 |
| EC025 | soil | Secondary forest | 200209 | 200209 | 200209 | 200209 | 200126 | 200126 | 171160 | 168545 | 110354 | 105982 | 102657 |
| EC026 | soil | Secondary forest | 97239 | 97239 | 97239 | 97239 | 97224 | 97224 | 80637 | 79788 | 52491 | 52140 | 51167 |
| EC027 | soil | Plantation forest | 125473 | 125473 | 125473 | 125473 | 125439 | 125439 | 102054 | 98925 | 62531 | 61602 | 59183 |
| EC028 | soil | Plantation forest | 96071 | 96071 | 96071 | 96071 | 96052 | 96052 | 78898 | 77006 | 49666 | 49242 | 48573 |
| EC029 | soil | Plantation forest | 124928 | 124928 | 124928 | 124928 | 124889 | 124889 | 114476 | 113447 | 83207 | 80925 | 77704 |
| EC030 | soil | Plantation forest | 123314 | 123314 | 123314 | 123314 | 123263 | 123263 | 113362 | 112906 | 82984 | 70971 | 67621 |
| EC031 | soil | Plantation forest | 76257 | 76257 | 76257 | 76257 | 76244 | 76244 | 66786 | 65660 | 44585 | 44185 | 43275 |
| EC037 | litter | Secondary forest | 111584 | 111584 | 111584 | 111584 | 111555 | 111555 | 95391 | 92276 | 57925 | 57475 | 57216 |
| EC038 | litter | Secondary forest | 177266 | 177266 | 177266 | 177266 | 177222 | 177222 | 149787 | 146995 | 93831 | 93313 | 92675 |
| EC039 | litter | Secondary forest | 146702 | 146702 | 146702 | 146702 | 146674 | 146674 | 125124 | 121518 | 77336 | 76598 | 76165 |
| EC040 | litter | Secondary forest | 98352 | 98352 | 98352 | 98352 | 98326 | 98326 | 78206 | 74724 | 41120 | 40397 | 40397 |
| EC041 | litter | Secondary forest | 140413 | 140413 | 140413 | 140413 | 140379 | 140379 | 116749 | 115912 | 69879 | 68191 | 67647 |
| EC042 | litter | Plantation forest | 155714 | 155714 | 155714 | 155714 | 155669 | 155669 | 121198 | 116127 | 64476 | 63037 | 62054 |
| EC043 | litter | Plantation forest | 68361 | 68361 | 68361 | 68361 | 68344 | 68344 | 53220 | 50814 | 30357 | 29779 | 29563 |
| EC044 | litter | Plantation forest | 85023 | 85023 | 85023 | 85023 | 84999 | 84999 | 67597 | 65391 | 36529 | 34816 | 34673 |
| EC045 | litter | Plantation forest | 162746 | 162746 | 162746 | 162746 | 162690 | 162690 | 126298 | 121142 | 67448 | 66157 | 65531 |
| EC046 | litter | Plantation forest | 221698 | 221698 | 221698 | 221698 | 221640 | 221640 | 191424 | 184525 | 112466 | 107905 | 106332 |
